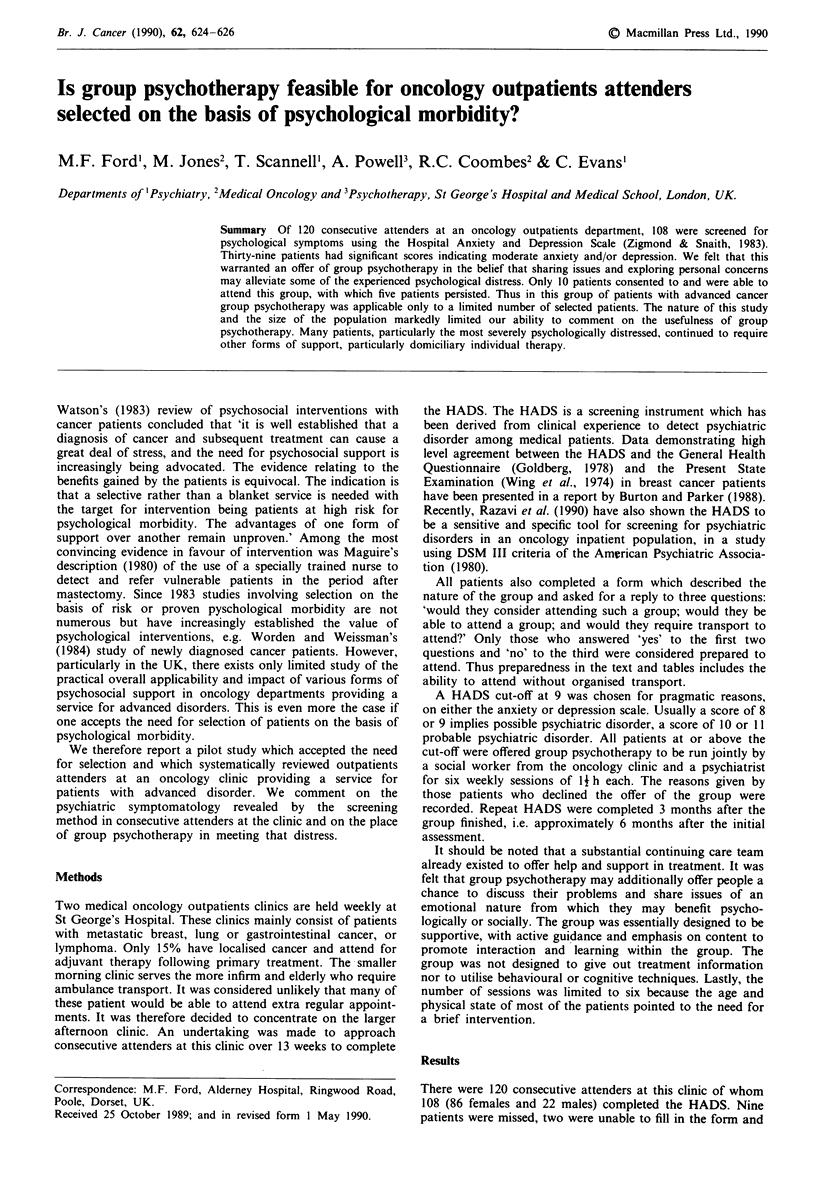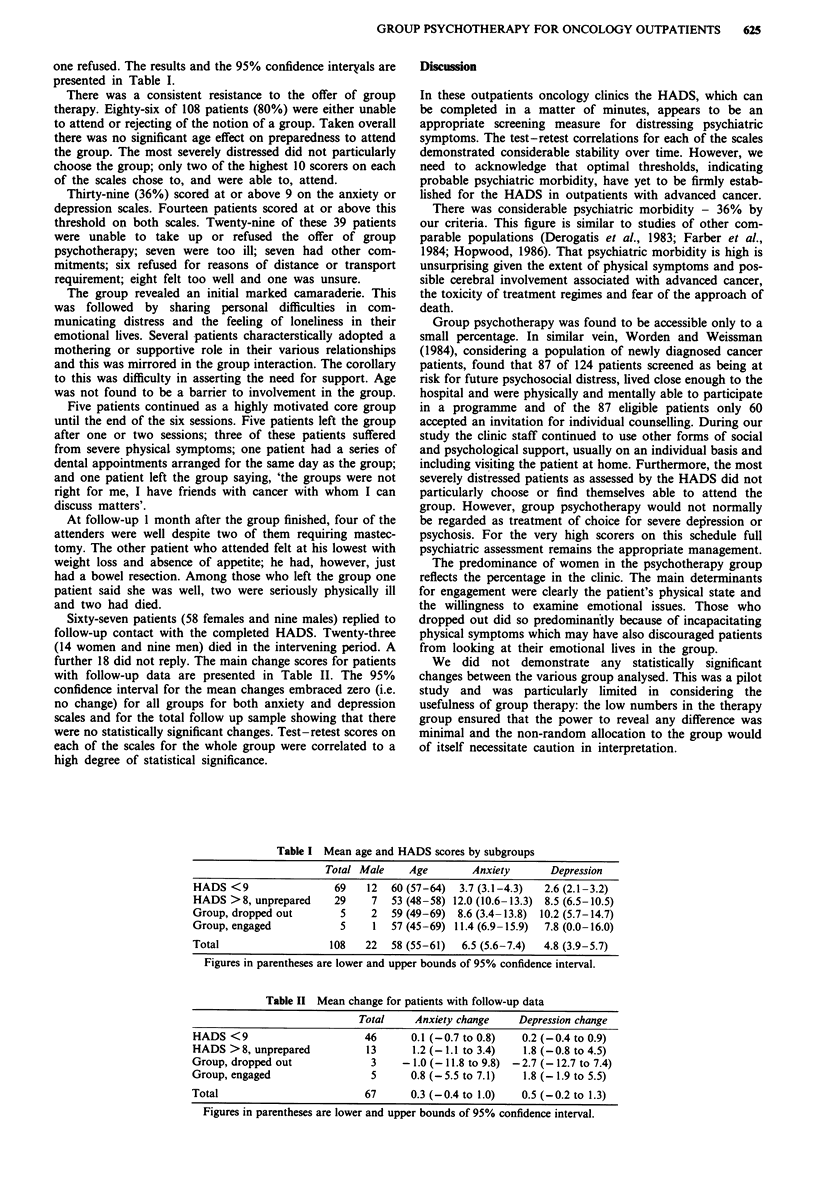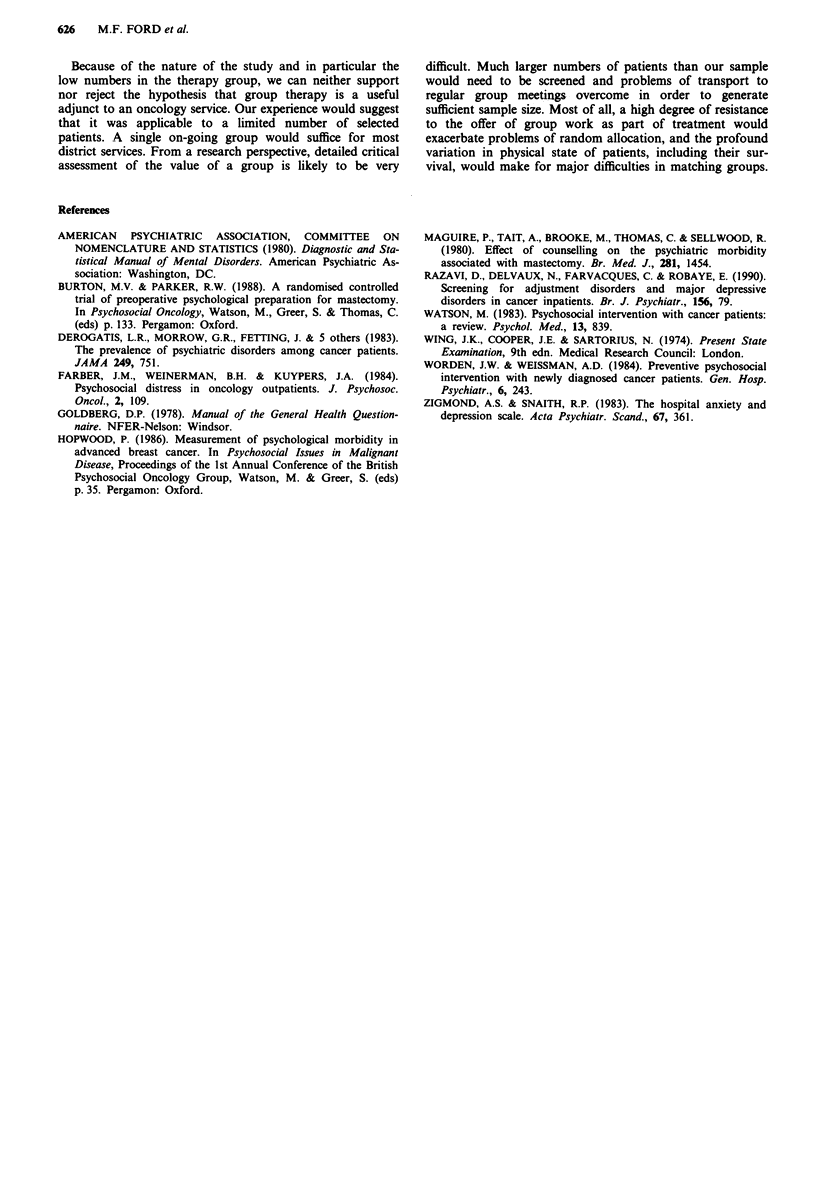# Is group psychotherapy feasible for oncology outpatients attenders selected on the basis of psychological morbidity?

**DOI:** 10.1038/bjc.1990.342

**Published:** 1990-10

**Authors:** M. F. Ford, M. Jones, T. Scannell, A. Powell, R. C. Coombes, C. Evans

**Affiliations:** Department of Psychiatry, St George's Hospital and Medical School, London, UK.

## Abstract

Of 120 consecutive attenders at an oncology outpatients department, 108 were screened for psychological symptoms using the Hospital Anxiety and Depression Scale (Zigmond & Snaith, 1983). Thirty-nine patients had significant scores indicating moderate anxiety and/or depression. We felt that this warranted an offer of group psychotherapy in the belief that sharing issues and exploring personal concerns may alleviate some of the experienced psychological distress. Only 10 patients consented to and were able to attend this group, with which five patients persisted. Thus in this group of patients with advanced cancer group psychotherapy was applicable only to a limited number of selected patients. The nature of this study and the size of the population markedly limited our ability to comment on the usefulness of group psychotherapy. Many patients, particularly the most severely psychologically distressed, continued to require other forms of support, particularly domiciliary individual therapy.


Br. J. Cancer (1990), 62, 624-626 © Macmillan Press Ltd., 1990

### Is group psychotherapy feasible for oncology outpatients attenders selected on the basis of psychological morbidity?

M.F. Ford[1], M. Jones[2], T. Scannell[1], A. Powell[3], R.C. Coombes[2] & C. Evans[1]

*Departments of [1]Psychiatry, [2]Medical Oncology and [3]Psychotherapy, St George's Hospital and Medical School, London, UK.*

**Summary** Of 120 consecutive attenders at an oncology outpatients department, 108 were screened for psychological symptoms using the Hospital Anxiety and Depression Scale (Zigmond & Snaith, 1983). Thirty-nine patients had significant scores indicating moderate anxiety and/or depression. We felt that this warranted an offer of group psychotherapy in the belief that sharing issues and exploring personal concerns may alleviate some of the experienced psychological distress. Only 10 patients consented to and were able to attend this group, with which five patients persisted. Thus in this group of patients with advanced cancer group psychotherapy was applicable only to a limited number of selected patients. The nature of this study and the size of the population markedly limited our ability to comment on the usefulness of group psychotherapy. Many patients, particularly the most severely psychologically distressed, continued to require other forms of support, particularly domiciliary individual therapy.

Watson's (1983) review of psychosocial interventions with cancer patients concluded that 'it is well established that a diagnosis of cancer and subsequent treatment can cause a great deal of stress, and the need for psychosocial support is increasingly being advocated. The evidence relating to the benefits gained by the patients is equivocal. The indication is that a selective rather than a blanket service is needed with the target for intervention being patients at high risk for psychological morbidity. The advantages of one form of support over another remain unproven.' Among the most convincing evidence in favour of intervention was Maguire's description (1980) of the use of a specially trained nurse to detect and refer vulnerable patients in the period after mastectomy. Since 1983 studies involving selection on the basis of risk or proven pyschological morbidity are not numerous but have increasingly established the value of psychological interventions, e.g. Worden and Weissman's (1984) study of newly diagnosed cancer patients. However, particularly in the UK, there exists only limited study of the practical overall applicability and impact of various forms of psychosocial support in oncology departments providing a service for advanced disorders. This is even more the case if one accepts the need for selection of patients on the basis of psychological morbidity.

We therefore report a pilot study which accepted the need for selection and which systematically reviewed outpatients attenders at an oncology clinic providing a service for patients with advanced disorder. We comment on the psychiatric symptomatology revealed by the screening method in consecutive attenders at the clinic and on the place of group psychotherapy in meeting that distress.

### Methods

Two medical oncology outpatients clinics are held weekly at St George's Hospital. These clinics mainly consist of patients with metastatic breast, lung or gastrointestinal cancer, or lymphoma. Only 15% have localised cancer and attend for adjuvant therapy following primary treatment. The smaller morning clinic serves the more infirm and elderly who require ambulance transport. It was considered unlikely that many of these patient would be able to attend extra regular appointments. It was therefore decided to concentrate on the larger afternoon clinic. An undertaking was made to approach consecutive attenders at this clinic over 13 weeks to complete the HADS. The HADS is a screening instrument which has been derived from clinical experience to detect psychiatric disorder among medical patients. Data demonstrating high level agreement between the HADS and the General Health Questionnaire (Goldberg, 1978) and the Present State Examination (Wing *et al*., 1974) in breast cancer patients have been presented in a report by Burton and Parker (1988). Recently, Razavi *et al*. (1990) have also shown the HADS to be a sensitive and specific tool for screening for psychiatric disorders in an oncology inpatient population, in a study using DSM III criteria of the American Psychiatric Association (1980).

All patients also completed a form which described the nature of the group and asked for a reply to three questions: 'would they consider attending such a group; would they be able to attend a group; and would they require transport to attend?' Only those who answered 'yes' to the first two questions and 'no' to the third were considered prepared to attend. Thus preparedness in the text and tables includes the ability to attend without organised transport.

A HADS cut-off at 9 was chosen for pragmatic reasons, on either the anxiety or depression scale. Usually a score of 8 or 9 implies possible psychiatric disorder, a score of 10 or 11 probable psychiatric disorder. All patients at or above the cut-off were offered group psychotherapy to be run jointly by a social worker from the oncology clinic and a psychiatrist for six weekly sessions of 1½ h each. The reasons given by those patients who declined the offer of the group were recorded. Repeat HADS were completed 3 months after the group finished, i.e. approximately 6 months after the initial assessment.

It should be noted that a substantial continuing care team already existed to offer help and support in treatment. It was felt that group psychotherapy may additionally offer people a chance to discuss their problems and share issues of an emotional nature from which they may benefit psychologically or socially. The group was essentially designed to be supportive, with active guidance and emphasis on content to promote interaction and learning within the group. The group was not designed to give out treatment information nor to utilise behavioural or cognitive techniques. Lastly, the number of sessions was limited to six because the age and physical state of most of the patients pointed to the need for a brief intervention.

### Results

There were 120 consecutive attenders at this clinic of whom 108 (86 females and 22 males) completed the HADS. Nine patients were missed, two were unable to fill in the form and

Correspondence: M.F. Ford, Alderney Hospital, Ringwood Road, Poole, Dorset, UK.
Received 25 October 1989; and in revised form 1 May 1990.

one refused. The results and the 95% confidence intervals are presented in Table I.

There was a consistent resistance to the offer of group therapy. Eighty-six of 108 patients (80%) were either unable to attend or rejecting of the notion of a group. Taken overall there was no significant age effect on preparedness to attend the group. The most severely distressed did not particularly choose the group; only two of the highest 10 scorers on each of the scales chose to, and were able to, attend.

Thirty-nine (36%) scored at or above 9 on the anxiety or depression scales. Fourteen patients scored at or above this threshold on both scales. Twenty-nine of these 39 patients were unable to take up or refused the offer of group psychotherapy; seven were too ill; seven had other commitments; six refused for reasons of distance or transport requirement; eight felt too well and one was unsure.

The group revealed an initial marked camaraderie. This was followed by sharing personal difficulties in communicating distress and the feeling of loneliness in their emotional lives. Several patients characterstically adopted a mothering or supportive role in their various relationships and this was mirrored in the group interaction. The corollary to this was difficulty in asserting the need for support. Age was not found to be a barrier to involvement in the group.

Five patients continued as a highly motivated core group until the end of the six sessions. Five patients left the group after one or two sessions; three of these patients suffered from severe physical symptoms; one patient had a series of dental appointments arranged for the same day as the group; and one patient left the group saying, 'the groups were not right for me, I have friends with cancer with whom I can discuss matters'.

At follow-up 1 month after the group finished, four of the attenders were well despite two of them requiring mastectomy. The other patient who attended felt at his lowest with weight loss and absence of appetite; he had, however, just had a bowel resection. Among those who left the group one patient said she was well, two were seriously physically ill and two had died.

Sixty-seven patients (58 females and nine males) replied to follow-up contact with the completed HADS. Twenty-three (14 women and nine men) died in the intervening period. A further 18 did not reply. The main change scores for patients with follow-up data are presented in Table II. The 95% confidence interval for the mean changes embraced zero (i.e. no change) for all groups for both anxiety and depression scales and for the total follow up sample showing that there were no statistically significant changes. Test–retest scores on each of the scales for the whole group were correlated to a high degree of statistical significance.

### Discussion

In these outpatients oncology clinics the HADS, which can be completed in a matter of minutes, appears to be an appropriate screening measure for distressing psychiatric symptoms. The test–retest correlations for each of the scales demonstrated considerable stability over time. However, we need to acknowledge that optimal thresholds, indicating probable psychiatric morbidity, have yet to be firmly established for the HADS in outpatients with advanced cancer.

There was considerable psychiatric morbidity – 36% by our criteria. This figure is similar to studies of other comparable populations (Derogatis *et al.*, 1983; Farber *et al.*, 1984; Hopwood, 1986). That psychiatric morbidity is high is unsurprising given the extent of physical symptoms and possible cerebral involvement associated with advanced cancer, the toxicity of treatment regimes and fear of the approach of death.

Group psychotherapy was found to be accessible only to a small percentage. In similar vein, Worden and Weissman (1984), considering a population of newly diagnosed cancer patients, found that 87 of 124 patients screened as being at risk for future psychosocial distress, lived close enough to the hospital and were physically and mentally able to participate in a programme and of the 87 eligible patients only 60 accepted an invitation for individual counselling. During our study the clinic staff continued to use other forms of social and psychological support, usually on an individual basis and including visiting the patient at home. Furthermore, the most severely distressed patients as assessed by the HADS did not particularly choose or find themselves able to attend the group. However, group psychotherapy would not normally be regarded as treatment of choice for severe depression or psychosis. For the very high scorers on this schedule full psychiatric assessment remains the appropriate management.

The predominance of women in the psychotherapy group reflects the percentage in the clinic. The main determinants for engagement were clearly the patient's physical state and the willingness to examine emotional issues. Those who dropped out did so predominantly because of incapacitating physical symptoms which may have also discouraged patients from looking at their emotional lives in the group.

We did not demonstrate any statistically significant changes between the various group analysed. This was a pilot study and was particularly limited in considering the usefulness of group therapy: the low numbers in the therapy group ensured that the power to reveal any difference was minimal and the non-random allocation to the group would of itself necessitate caution in interpretation.

**Table I** Mean age and HADS scores by subgroups

| | *Total* | *Male* | *Age* | *Anxiety* | *Depression* |
|---|---|---|---|---|---|
| HADS <9 | 69 | 12 | 60 (57–64) | 3.7 (3.1–4.3) | 2.6 (2.1–3.2) |
| HADS >8, unprepared | 29 | 7 | 53 (48–58) | 12.0 (10.6–13.3) | 8.5 (6.5–10.5) |
| Group, dropped out | 5 | 2 | 59 (49–69) | 8.6 (3.4–13.8) | 10.2 (5.7–14.7) |
| Group, engaged | 5 | 1 | 57 (45–69) | 11.4 (6.9–15.9) | 7.8 (0.0–16.0) |
| Total | 108 | 22 | 58 (55–61) | 6.5 (5.6–7.4) | 4.8 (3.9–5.7) |

Figures in parentheses are lower and upper bounds of 95% confidence interval.

**Table II** Mean change for patients with follow-up data

| | *Total* | *Anxiety change* | *Depression change* |
|---|---|---|---|
| HADS <9 | 46 | 0.1 (−0.7 to 0.8) | 0.2 (−0.4 to 0.9) |
| HADS >8, unprepared | 13 | 1.2 (−1.1 to 3.4) | 1.8 (−0.8 to 4.5) |
| Group, dropped out | 3 | −1.0 (−11.8 to 9.8) | −2.7 (−12.7 to 7.4) |
| Group, engaged | 5 | 0.8 (−5.5 to 7.1) | 1.8 (−1.9 to 5.5) |
| Total | 67 | 0.3 (−0.4 to 1.0) | 0.5 (−0.2 to 1.3) |

Figures in parentheses are lower and upper bounds of 95% confidence interval.

Because of the nature of the study and in particular the low numbers in the therapy group, we can neither support nor reject the hypothesis that group therapy is a useful adjunct to an oncology service. Our experience would suggest that it was applicable to a limited number of selected patients. A single on-going group would suffice for most district services. From a research perspective, detailed critical assessment of the value of a group is likely to be very difficult. Much larger numbers of patients than our sample would need to be screened and problems of transport to regular group meetings overcome in order to generate sufficient sample size. Most of all, a high degree of resistance to the offer of group work as part of treatment would exacerbate problems of random allocation, and the profound variation in physical state of patients, including their survival, would make for major difficulties in matching groups.

**References**

AMERICAN PSYCHIATRIC ASSOCIATION, COMMITTEE ON NOMENCLATURE AND STATISTICS (1980). *Diagnostic and Statistical Manual of Mental Disorders*. American Psychiatric Association: Washington, DC.

BURTON, M.V. & PARKER, R.W. (1988). A randomised controlled trial of preoperative psychological preparation for mastectomy. In *Psychosocial Oncology*, Watson, M., Greer, S. & Thomas, C. (eds) p. 133. Pergamon: Oxford.

DEROGATIS, L.R., MORROW, G.R., FETTING, J. & 5 others (1983). The prevalence of psychiatric disorders among cancer patients. *JAMA* **249**, 751.

FARBER, J.M., WEINERMAN, B.H. & KUYPERS, J.A. (1984). Psychosocial distress in oncology outpatients. *J. Psychosoc. Oncol.*, **2**, 109.

GOLDBERG, D.P. (1978). *Manual of the General Health Questionnaire*. NFER-Nelson: Windsor.

HOPWOOD, P. (1986). Measurement of psychological morbidity in advanced breast cancer. In *Psychosocial Issues in Malignant Disease*, Proceedings of the 1st Annual Conference of the British Psychosocial Oncology Group, Watson, M. & Greer, S. (eds) p. 35. Pergamon: Oxford.

MAGUIRE, P., TAIT, A., BROOKE, M., THOMAS, C. & SELLWOOD, R. (1980). Effect of counselling on the psychiatric morbidity associated with mastectomy. *Br. Med. J.*, **281**, 1454.

RAZAVI, D., DELVAUX, N., FARVACQUES, C. & ROBAYE, E. (1990). Screening for adjustment disorders and major depressive disorders in cancer inpatients. *Br. J. Psychiatr.*, **156**, 79.

WATSON, M. (1983). Psychosocial intervention with cancer patients: a review. *Psychol. Med.*, **13**, 839.

WING, J.K., COOPER, J.E. & SARTORIUS, N. (1974). *Present State Examination*, 9th edn. Medical Research Council: London.

WORDEN, J.W. & WEISSMAN, A.D. (1984). Preventive psychosocial intervention with newly diagnosed cancer patients. *Gen. Hosp. Psychiatr.*, **6**, 243.

ZIGMOND, A.S. & SNAITH, R.P. (1983). The hospital anxiety and depression scale. *Acta Psychiatr. Scand.*, **67**, 361.